# Introducing a new chemically defined medium and feed for hybridoma cell lines

**DOI:** 10.1186/1753-6561-7-S6-P82

**Published:** 2013-12-04

**Authors:** Christoph Heinrich, Tim F Beckmann, Sandra Klausing, Stefanie Maimann, Bernd Schröder, Stefan Northoff

**Affiliations:** 1 TeutoCell AG, Bielefeld, 33613, Germany; 2 Miltenyi Biotec GmbH, Teterow, 17166, Germany

## Background

Hybridoma technology was established in the 2nd half of the 20th century and in the view of current protein production it might seem old-fashioned. Despite, it is commonly used to produce monoclonal antibodies (mAbs) for R & D, clinical diagnostics or medical applications and the demand for mAbs produced by hybridomas is still high. However, compared to CHO, only a few serum-free hybridoma media are available and even less suppliers for chemically defined products are on the market. In this work, a new chemically defined medium and feed were developed to bring hybridoma processes to the next level and to target the existing gap in the market.

## Materials and methods

HybriMACS CD medium was developed using various research and production hybridoma cell lines from Bielefeld University (e.g. MF20, 187.1, HB8209) and industrial partners. HybriMACS CD medium was supplemented with 8 mM L-Glutamine for routine cultivation, batch and perfusion processes. For optimal performance of MF20 hybridoma cells (DSHB at the University of Iowa) the HybriMACS CD medium was supplemented with insulin (4 mg/L) or IGF (0.04 mg/L).

All cultivations were carried out using standard conditions. Briefly, precultures and batch cultivations were performed in 125 mL and 250 mL Erlenmeyer flasks. Incubator conditions were set to 37 °C, 5% CO_2 _and a relative humidity of 80%. For bioreactor cultivations closed-loop controlled 2 L benchtop systems were used with parameters set to 37 °C, 40% DO and pH 7.1 +/- 0.05. Automated viable cell counting was performed using a Cedex (Innovatis). Monoclonal antibody (mAb) concentrations were determined with Protein A HPLC or ELISA (MF20 cell line).

## Results

**Figure 1:**
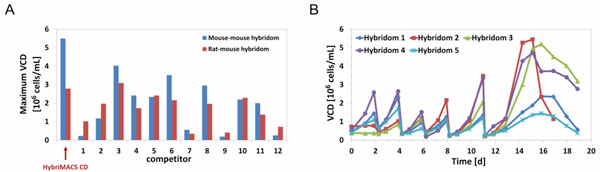
**(A) Comparison of two different hybridoma cell lines in HybriMACS CD and 12 competitor media**. **(B) **Growth behaviour of five serum-dependent hybridoma cell lines in HybriMACS CD directly after thawing.

Hybridoma cell growth in HybriMACS CD medium was compared to 12 competitor products in the time course of several passages and a final batch cultivation. For a mouse-mouse hybridoma cell line, maximum viable cell density (vcd) in HybriMACS CD was highest and for a rat-mouse hybridoma cell line second-highest compared to growth in the 12 competitor media, as shown in Figure 1 (A).

Easy adaption from serum-containing medium was verified by direct thawing of five different hybridoma cell lines in HybriMACS CD (Figure 1 B). Furthermore, long-term stable growth of a hybridoma cell line in HybriMACS CD was also confirmed in cultivations for more than 80 days.

The majority of tested cell lines reached a maximum cell density above 2.5 to 5.0 × 10^6 ^cells/mL in uncontrolled and controlled batch processes using HybriMACS CD. For uncontrolled fed batch cultivations 1.0 × 10^7 ^cells/mL were observed as maximum viable cell density, while controlled fed batch processes reached values above 1.5 × 10^7 ^cells/mL. The final antibody titer was increased at least by a factor of 5 in uncontrolled fed batches and up to 10 times in controlled fed batch cultivations using HybriMACS Feed Supplement. Exemplary results of controlled as well as uncontrolled batch and fed batch cultivations are shown in Table 1.

**Table 1:** Exemplary data of batch and fed batch cultivations under controlled (bioreactor) as well as uncontrolled (shaker) conditions

	Process	Maximum vcd [10^6 ^cells/mL]	IVCD [10^6 ^(cells*d)/mL]	Final mAb titer [mg/L]
**Shaker**	**Batch**	3.7	7.3	56.2

	**Fed batch**	10.5	39.5	447.5

**Bioreactor**	**Batch**	3.7	9.52	61.0

	**Fed batch**	17.3	107.6	1035.4

Values represent mean from two biological replicates.

## Conclusions

HybriMACS CD is a chemically-defined, protein-free medium composition with no need for growth hormone supplementation. The specially designed formulation supports direct adaption of serum-dependent hybridoma cells, even when starting from a serum-containing cell bank. In addition, the developed medium formulation enables stable long-term growth of hybridoma cell lines, supporting an unrestricted utilization in diverse processes. HybriMACS CD is suitable for bioreactor batch and perfusion processes reaching high cell densities and commonly accepted amounts of antibody. A specially tailored HybriMACS Feed Supplement increased final antibody titer at least by a factor of 5 to 10 for all tested hybridoma cell lines. This improvement can be further increased by customization of the generic feed regime, while maintaining suitable glucose and glutamine concentrations.

